# Refractive and visual outcomes of plate-haptic toric intraocular lens implantation for astigmatic correction after uncomplicated microincision cataract surgery

**DOI:** 10.22336/rjo.2022.57

**Published:** 2022

**Authors:** Shalini Kumari, Harbansh Lal, Tinku Bali Razdan, Mohini Agrawal, Nitin Vichare

**Affiliations:** * Department of Ophthalmology, Command Hospital, Pune, India; ** Department of Ophthalmology, Sir Ganga Ram Hospital, New Delhi, India

**Keywords:** plate-haptic toric, microincision, astigmatism, cataract surgery

## Abstract

**Objective:** To evaluate visual and refractive outcomes with plate-haptic toric intraocular lens implantation (ph-toric) after uncomplicated microincision cataract surgery (MICS) to correct moderate-to-severe astigmatism.

**Design:** Prospective cross-sectional study.

**Methods:** The study was conducted at a tertiary eye care center in New Delhi, India, on patients with visually-significant cataract and moderate-to-severe astigmatism (>1.00 diopters [D]). Preoperative parameters like visual-acuity, keratometry and astigmatism values and lens power calculation were noted. After MICS via 1.8 mm incision, a ph-toric IOL was implanted. The outcome measures noted were uncorrected and corrected distant visual-acuity (UDVA, CDVA), decrease in astigmatism and rotational stability. Follow-up was done on day 1, day 7, 1-month and 3-months postoperative.

**Results:** This study involved 30 eyes of 30 patients. 27 patients (90%) gained UDVA of 6/ 9 or better. Out of these, 27 patients (90%) achieved CDVA of 6/ 6. Mean CDVA changed from 0.967 ± 0.101 postoperative to 0.176 ± 0.82 preoperatively (p<0.001). The mean preoperative astigmatism was 2.08 ± 0.59 D and the mean postoperative astigmatism was 0.35 ± 0.39 D. The mean correction achieved was 1.28 ± 0.32 D. Statistically significant (p<0.001) correction of astigmatism was observed by use of ph-toric IOL. The mean reduction in astigmatism was 84.16 ± 10.61 with excellent reduction in 43.3%. IOL rotation was <10 degrees in all the eyes. No complications were observed. Patients had satisfaction with the procedure and visual outcomes.

**Conclusion:** Implantation of a plate-haptic toric IOL after uncomplicated MICS via 1.8 mm incision is a feasible and safe option in cataract cases with astigmatism to provide good visual and refractive outcomes. No major drawbacks were observed attributable to ph-toric IOL.

**Abbreviations:** SIA - surgically induced astigmatism, D - diopters, MICS - microincision cataract surgery, ph-toric IOL - plate-haptic toric IOL, IOL - intraocular lens, UDVA - uncorrected distant visual acuity, CDVA - corrected distant visual acuity

## Introduction

At present, the goal of every cataract surgeon is to achieve an excellent visual outcome postoperatively with minimum induction of refractive-error, astigmatism; therefore, providing a good visual rehabilitation. Correction of astigmatic errors and control of surgically induced astigmatism (SIA) are now an essential part of such operative procedures. Astigmatic errors are visually-disabling errors of refraction that affect the general population. A total of 15-to-20% of the cases with cataract have ≥ 1.50 diopters (D) of corneal astigmatism [**1**,**2**]. 

Improvements in pre-existing and SIA astigmatic outcomes are now possible by focusing upon and obtaining more accurate preoperative cylindrical measurements, careful surgical planning particularly regarding incision(s), design, suture-less and use of toric IOLs [**3**,**4**].

In today’s eon, the most recent advances in phacoemulsification is microincision cataract surgery (MICS). It is a surgery performed via incisions of < 2.0 mm [**5**]. The astigmatically neutral MICS incisions lessen SIA and surgical trauma. This causes improved quality of corneal optical surface and better visual outcomes with spectacle independence. On top of that, the use of toric IOLs in MICS can do wonders in effective astigmatic correction [**6**]. Toric IOL implantation is a predictable, constant and safe mode to lessen pre-existing astigmatism.

Indian literature is scarce in reporting the efficacy of plate-haptic toric IOL (ph-toric IOL) in MICS. Thus, the aim of this study was to evaluate visual outcomes, refractive outcomes, drop in astigmatism, rotational stability and safety post MICS with ph-toric IOL implantation in cataract patients with mild-to-severe astigmatism.

## Material and methods

This prospective non-randomized study included consecutive patients with visually significant cataract and astigmatism between 1.00 and 4.00 D. They were planned for MICS with ph-toric IOL implantation from November 2018 to April 2019. Surgery was performed on the same day by a single surgeon. It was approved by the Institutional Ethics Committee and conducted in accordance with the Declaration of Helsinki. Patients were explained the risks and complications of the study and a written informed consent was signed by all of them.

Patients of the age group between 55 to 80 years, with regular astigmatism ranging from 1.0 D to 4.0 D and nuclear sclerosis of grade I, II and III (based on Lens Opacities Classification System-III), were included in our study. Exclusion criteria included irregular astigmatism, lenticular astigmatism, previous refractive or intraocular surgery, glaucoma; fundus pathologies like retinopathy, maculopathy, retinal detachment; media opacity other than cataract (cornea or vitreous), uveitis or any corneal endothelial disease. Also, patients with any intraoperative complications were also excluded from the study. 

A detailed ophthalmological examination was done in all the patients. Preoperative examination like uncorrected distant visual acuity (UDVA) and corrected distant visual acuity (CDVA) using Snellen charts along with manifest and cycloplegic refraction, slit-lamp examination, intraocular pressures by Goldmann applanation tonometry, fundus examination and corneal topography were performed. Keratometry was performed using Bausch and Lomb type keratometer, Canon autorefractometer and Zeiss IOL master). Biometry was done using Zeiss IOL master. All the required values were referred to the IOL manufacturer who calculated the IOL power and axis to attain emmetropia.

A ph-toric IOL (Zeiss) was implanted in all eyes. A bi-toric, single-piece, ph, foldable acrylic aspheric IOL with water-content of 25% was used. It was available with an optic diameter of 6.0 mm, having an overall size of 11.0 mm, refractive index 1.46, central thickness of 0.7 mm and edge thickness of 0.25 to 0.27 mm. 

An informed consent was obtained and patients were planned for MICS via 1.8 mm incision with ph-toric IOL implantation. Pupils were dilated with a solution of tropicamide and phenylephrine. Preoperatively, reference marks were made on the limbus at the 0 and 180-degree position with the patient seated upright in front of the slit-lamp. All patients underwent the surgery under peribulbar anesthesia using the same technique via 1.8 mm incision. Additional toric markings were made on the operation table representing the steep corneal axis using a Mendez degree gauge.

All MICS surgeries were performed by a single surgeon in a standardized fashion, via 1.8 mm clear corneal incision. Continuous curvilinear capsulorhexis was performed followed by hydro-procedures. Phacoemulsification of the nucleus was done using divide and conquer technique followed by IOL implantation. The ph-toric IOL was put in-the-bag by making a 1.8 mm incision. A viso-dispersive was used in all the cases. After the complete removal of the viscoelastic substance, the ph-IOL was rotated to align with the axis marked as reference marks. All cataract surgeries were performed by longitudinal mode of phacoemulsification using one type of ph-IOL, one type of intraocular irrigation solution and one type of viscoelastic material. 

After the removal of the viscoelastic substance, stromal hydration of all the incisions was done. Subconjunctival antibiotic and steroid were administered at the end of the surgery. Applied eye patch was removed on the next day and patients were started on topical antibiotics and steroids in tapering doses for a month.

All the patients underwent post-operative ocular examination and parameters like UDVA, CDVA, refraction, keratometry, alignment of the ph-toric IOL were noted in the follow-up period on day 1, day 7, 1-month and 3-months. All the evaluations were performed by the same surgeon using a similar method. The ph-toric axis was calculated by aligning a thin coaxial slit with the IOL axis marked with a slit-lamp. The extent of correction was divided into four grades: excellent, good, fair and poor, based on the decrease in astigmatism from the preoperative data as >90%, 80-90%, 70-79% and <70%, respectively.

Data was analyzed using SPSS for Windows (version 17.0). Qualitative data variables were expressed as frequency and percentage while quantitative data variables were expressed as Mean and SD. Paired t-test was applied to see the difference between different groups. A confidence level of 95% and p-value <0.05 were considered significant.

## Results

Thirty eyes of 30 consecutive patients were included in the study. The mean age of presentation was 59.20 ± 5.47years. 17 (56.7%) patients were aged between 50 to 60 years and the rest of 13 (43.3%) were aged between 60 to 70 years. The demographics of patients are depicted in **Table 1** along with their preoperative refractive and keratometry data. 

**Table 1:** Patient demographics and preoperative data

Characteristic		Value
Patients		30
Eyes (n)		30
Age (years)		
	Mean ± SD	59.20 ± 5.47
Sex (n,%)		
	Males	21 (70)
	Females	09 (30)
Preoperative data		
Keratometry (D)		
K1	Mean ± SD (Range)	42.85 ± 1.65 (38.87 - 45.53)
K2	Mean ± SD (Range)	44.97 ± 1.72 (41.24 - 48.98)
Axial length (mm)		
	Mean ± SD	23.89 ± 2.73
	Range	20.21 to 28.55
IOL power (D)		
	Range	16-25

**Table 2 A**, **B** shows the pre-operative and post-operative keratometric values and astigmatic values of all the eyes. The mean pre-operative corneal astigmatism was 2.08 ± 0.59 D and the mean postoperative corneal astigmatism was 0.35 ± 0.39 D. The mean correction by ph-toric IOL was 1.28 ± 0.32 D. There was a statistically high significant correction of astigmatism (p<0.001, t test) using ph-toric IOL in this study.

**Table 2 A:** Preoperative and postoperative values of keratometry and astigmatism of all 30 eyes

Patient	Preoperative		Postoperative		Astigmatism	
	K1	K2	K1	K2	Preoperative	Postoperative
1	43.32	45.18	43.34	43.02	1.86 x 180	0.32 x 178
2	43.38	46.36	43.40	43.58	2.48 x 100	0.18 x 98
3	41.21	42.88	41.24	41.28	1.67 x 145	0.04 x 140
4	43.05	44.47	43.10	43.22	1.42 x 90	0.12 x 90
5	39.74	41.24	39.74	40.18	1.50 x 90	0.44 x 92
6	39.7	42.6	39.74	40.18	2.90 x 90	0.44 x 88
7	38.87	41.92	38.96	38.96	3.05 x 145	0.90 x 142
8	43.65	44.81	43.69	43.89	1.16 x 180	0.20 x 181
9	43.6	46.00	43.69	44.04	2.4 x 170	0.45 x 174
10	42.43	44.55	42.49	43.68	2.12 x 90	0.18 x 92
11	45.53	46.78	45.54	45.68	1.25 x 100	0.14 x 98
12	44.26	45.22	44.34	44.62	1.66 x 155	0.28 x 158
13	44.18	46.36	44.21	44.28	2.18 x 100	0.07 x 98
14	42.21	43.98	42.24	42.28	1.67 x 142	0.04 x 146
15	44.68	46.57	44.72	44.81	1.89 x 78	0.09 x 76
16	42.05	44.37	42.10	42.27	1.42 x 68	0.11 x 66
17	45.18	48.98	45.24	46.68	3.80 x 170	1.44 x 164
18	43.40	45.70	43.49	44.28	2.30 x 170	0.79 x 174
19	40.91	42.83	40.94	41.24	1.92 x 90	0.30 x 86
20	44.12	46.24	44.14	44.54	2.12 x 95	0.40 x 98
21	43.38	46.36	43.40	44.30	2.98 x 170	0.90 x 172
22	41.11	43.35	41.14	41.28	1.92 x 168	0.27 x 164
23	42.99	44.64	43.01	44.41	1.65 x 157	0.40 x 160
24	43.36	45.68	43.48	43.36	2.32 x 99	0.12 x 102
25	40.96	42.70	41.00	41.13	1.84 x 97	0.13 x 94
26	42.49	44.83	42.52	42.63	2.34 x 104	0.12 x 102
27	43.67	45.47	43.69	43.70	1.80 x 142	0.04 x 140
28	44.36	46.24	44.42	44.51	1.88 x 170	0.09 x 168
29	43.49	45.53	43.52	43.58	2.04 x 104	0.06 x 100
30	44.36	47.18	44.38	44.98	2.82 x 168	1.60 x 166
Mean ± S D	42.85 ± 1.65	44.97 ± 1.72	42.90 ± 1.66	43.22 ± 1.76	2.08 ± 0.59	0.35 ± 0.39

**Table 2 B:** Comparison analysis of keratometry values preoperatively and postoperatively in all eyes

	Preoperative		Postoperative		
	Mean ± SD	Range	Mean ± SD	Range	p-value
K1	42.85 ± 1.65	38.87 - 45.53	42.90 ± 1.66	38.96 - 45.54	<0.001
K2	44.97 ± 1.72	41.24 - 48.98	43.22 ± 1.76	38.96 - 46.68	<0.001

**Fig. 1:**
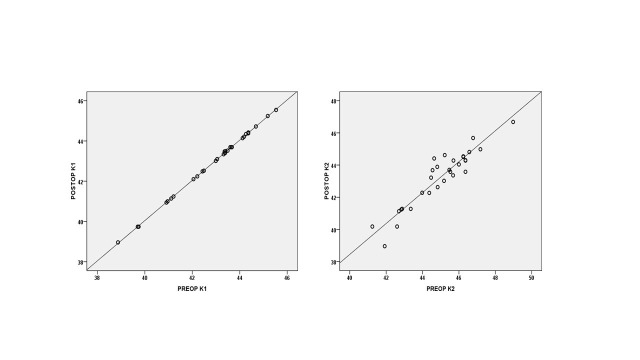
Scatterplot of mean preoperative versus postoperative refractive values depicting a positive correlation between preoperatively and postoperatively keratometry values (K1 and K2)

**Fig. 1** shows a perfect positive linear correlation between preoperative and postoperative K1 values (r=1.000, p<0.001) and a positive correlation between preoperative and postoperative K2, (r=0.937, p<0.001).

**Table 3** shows the surgical outcome of the patients after the surgical correction of astigmatism. The % of astigmatism corrected and success of astigmatism surgery was found to be “excellent” in 43.3% cases, followed by good results in 33.3%. Only 3 eyes had a poor extent of correction. The mean extent of correction was 84.16 ± 10.61.

**Table 3:** Surgical outcome after microincision surgery with plate-haptic toric intraocular lens implantation in all the patients

Patient	Preoperative astigmatism	Postoperative astigmatism	Reduction in astigmatism	Extent of correction
1	1.86 x 180	0.32 x 178	82.80%	Good
2	2.48 x 100	0.18 x 98	92.74%	Excellent
3	1.67 x 145	0.04 x 140	97.60%	Excellent
4	1.42 x 90	0.12 x 90	91.55%	Excellent
5	1.50 x 90	0.44 x 92	70.67%	Fair
6	2.90 x 90	0.44 x 88	84.83%	Good
7	3.05 x 145	0.90 x 142	70.49%	Fair
8	1.16 x 180	0.20 x 181	82.76%	Good
9	2.4 x 170	0.45 x 174	81.25%	Good
10	2.12 x 90	0.18 x 92	91.51%	Excellent
11	1.25 x 100	0.14 x 98	88.80%	Good
12	1.66 x 155	0.28 x 158	82.80%	Good
13	2.18 x 100	0.07 x 98	92.74%	Excellent
14	1.67 x 142	0.04 x 146	97.60%	Excellent
15	1.89 x 78	0.09 x 76	91.55%	Excellent
16	1.42 x 68	0.11 x 66	91.55%	Excellent
17	3.80 x 170	1.44 x 164	62.11%	Poor
18	2.30 x 170	0.79 x 174	65.65%	Poor
19	1.92 x 90	0.30 x 92	70.67%	Fair
20	2.12 x 95	0.40 x 98	84.83%	Good
21	2.98 x 170	0.90 x 172	70.49%	Fair
22	1.92 x 168	0.27 x 164	82.76%	Good
23	1.65 x 157	0.40 x 160	81.25%	Good
24	2.32 x 99	0.12 x 102	91.51%	Excellent
25	1.84 x 97	0.13 x 94	88.80%	Good
26	2.34 x 104	0.12 x 102	92.74%	Excellent
27	1.80 x 142	0.04 x 140	97.60%	Excellent
28	1.88 x 170	0.09 x 168	91.55%	Excellent
29	2.04 x 104	0.06 x 100	91.55%	Excellent
30	2.82 x 168	1.60 x 166	62.11%	Poor

The mean ph-toric IOL axis rotation was 1.75 ± 2 degrees (range 0-to-10) 3 months postoperatively. Only one eye had a rotation up to 10 degrees, all the had a rotation within 5 degrees. No eye had any significant rotation of the ph-toric IOL (>10 degrees). All the surgeries were uneventful intraoperatively, as well as postoperatively. None of the cases required IOL repositioning over 3-month follow-up. 

In this study, 27 cases (90%) gained UDVA of 6/ 12 or better. 28 cases (93.33%) achieved CDVA of 6/ 12 or better. Out of these 28, 27 cases (90%) gained vision to 6/ 6 with significant improvement in the postoperative vision of the patients (p<0.001) (**Table 4**). 

**Table 4:** Visual outcome at 3-month follow-up of all the patients after microincision cataract surgery with plate haptic-toric intraocular lens implantation

	Preoperative		Postoperative		
	Mean ± SD	Range	Mean ± SD	Range	p-value
Corrected distant visual acuity	0.176 ± 0.82	0.05 - 0.33	0.967 ± 0.101	0.67 - 1.00	<0.001

## Discussion

The contemporary concept of cataract surgery is inclined towards attaining the best corrected visual acuity postoperatively. The surgery should be safe, stable and precise. For patients with moderate-to-severe astigmatism, it is, nowadays, essential to look out for the astigmatism correction at the time of cataract surgery because cataract surgery has now become a refractive surgery in which the patients’ expectations are more demanding for full spectacle independence. 

Best vision is measured by taking care of both preexisting astigmatism and SIA [**7**]. Hence, in this study, MICS was performed with a smaller 1.8 mm incision (astigmatically neutral microincisions) combined with the implantation of ph-toric IOL (taking care of preexisting astigmatism).

MICS relates to additional wound stability, a decline in regular and irregular astigmatism and decline in corneal aberrations leading to enhanced visual outcomes and high patient gratification [**8**]. The ph-toric IOL corrects pre-existing astigmatism and easily fits via microincision without altering or compromising the optical quality of an IOL. To the best of our knowledge, this is the first study in India to evaluate visual and refractive outcomes of MICS via 1.8 mm incision with ph-oric IOL implantation in reducing moderate-to-severe astigmatism in patients with cataract. 

All the patients in our study showed satisfactory correction of DVA and astigmatism, which is in accordance with studies conducted by Alió et al. [**8**]. In the present study, post-operative UDVA showed statistically significant improvement as compared to preoperative DVA. UDVA of 6/ 12 or better was seen in 90% of the patients. There was no change in post-operative uncorrected distance visual acuity after two weeks. This data is consistent with other studies. In a previous study by Ruhswurm et al., 67.5% of the patients had a UDVA of 6/ 12 or better postoperatively [**9**]. De Silva et al. reported 78.6% of the patients with 6/ 12 or better UDVA [**10**]. Till et al. reported 10.66% of the patients with UDVA of better than 6/ 12 postoperatively [**11**].

In the present study, the mean pre-operative corneal astigmatism was 2.08 ± 0.59 D and the mean post-operative corneal astigmatism was 0.35 ± 0.39 D. The mean correction by toric IOL was 1.28 ± 0.32 D. In a study by Alió et al., the mean refractive cylinder decreased significantly after surgery from 4.46 D ± 2.23 to -0.45 ± 0.63 D [**12**,**13**]. Other studies on toric IOLs used incisions of ≥2.75 mm and the bigger incisions may affect the overall decrement in astigmatism post-operatively [**9**-**11**].

The placement of toric IOL in the correct axis is of utmost importance followed by IOL maintaining its position at that axis. This study found that keratometric assessment of astigmatism followed by the marking on the cornea preoperatively and on-the-table was adequate. Also, performing the cataract surgery via a smaller incision of 1.8 mm produced better results in terms of less SIA and better healing. A central 5.5 mm CCC ensured that the cornea remained central. An important step was the careful removal of all excess viscoelastic from the bag, to ensure that there was no rotation. 

The foremost necessity of any toric IOL is rotational stability in the capsular bag [**9**]. Often, toric IOL may rotate to some degree because of haptic compression from the capsular contraction, even after an uneventful cataract surgery. This change mostly arises in the first 3-months after the surgery. Thus, in this study the rotational stability of the ph-toric was assessed at 3-months post-operatively; none of the IOLs rotated >10 degrees. Every degree of misalignment is important as the corrected astigmatism reduces by 3.3% per degree rotation and there is a complete loss of IOL cylinder power correction when the IOL is misaligned by 30 degrees [**14**-**16**].

A previous study found that the IOLs with a C-loop-haptic had the maximum rate of postoperative rotation of >10 degrees (41%) [**17**]. Although other plate-haptic torics have better stability, they can still have a significant rotation (2% to 50%, >10 degrees). Our rotational outcomes were comparable to those in studies of the AcrySof torics. However, our results were rather better, since no IOL had >10 degrees of rotation. It was stable within the capsular-bag regarding rotation, which a requirement for toric IOLs. The ph-toric IOL used in MICS in this study fulfilled the requirements regarding foldability, not showing any evident impairment when injected with the MICS injector. No complications were associated to toric IOL implantation in our study. Regarding the patients’ satisfaction, they were satisfied with the combined procedure. Thus, MICS with ph-toric IOL is a safe, predictable and effective surgical choice to correct pre-existing corneal astigmatism during cataract surgery. Further studies with larger sample sizes and longer follow-ups are warranted. 

## Conclusion

MICS via 1.8 mm incision with plate-haptic toric IOL implantation is a feasible option for the correction of mild-to-severe astigmatism in patients with cataract. This combined procedure provides good visual and refractive outcomes after cataract surgery by countering preexisting, as well as SIA. The study did not expose any major drawbacks attributable to plate-haptic toric IOL. The quality of the corrected distance visual acuity with astigmatic correction was good, the patient achieving a good visual acuity.

**Conflict of Interest statement**

The authors state no conflict of interest.

**Informed Consent and Human and Animal Rights statement**

Informed consent has been obtained from all individuals included in this study.

**Authorization for the use of human subjects**

Ethical approval: The research related to human use complies with all the relevant national regulations, institutional policies, is in accordance with the tenets of the Helsinki Declaration, and has been approved by the Institutional Ethics Committee of Command Hospital, Pune, India. 

**Acknowledgements**

None.

**Sources of Funding**

None.

**Disclosures**

None.
